# Comparison of four in vitro test methods to assess nucleus pulposus replacement device expulsion risk

**DOI:** 10.1002/jsp2.1332

**Published:** 2024-04-23

**Authors:** Tamanna Rahman, Matthew J. Kibble, Gianluca Harbert, Nigel Smith, Erik Brewer, Thomas P. Schaer, Nicolas Newell

**Affiliations:** ^1^ Department of Bioengineering Imperial College London London UK; ^2^ Biomechanics Group, Department of Mechanical Engineering Imperial College London London UK; ^3^ Division of Surgery and Interventional Science University College London Stanmore UK; ^4^ Department of Biomedical Engineering Rowan University Glassboro New Jersey USA; ^5^ Department of Clinical Studies New Bolton Center University of Pennsylvania School of Veterinary Medicine Kennett Square Pennsylvania USA

**Keywords:** biomechanical testing, expulsion, hydrogel, intervertebral disk, nucleus replacement, spine

## Abstract

**Background:**

Nucleus replacement devices (NRDs) are not routinely used in clinic, predominantly due to the risk of device expulsion. Rigorous in vitro testing may enable failure mechanisms to be identified prior to clinical trials; however, current testing standards do not specify a particular expulsion test. Multiple methods have therefore been developed, complicating comparisons between NRD designs. Thus, this study assessed the effectiveness of four previously reported expulsion testing protocols; hula‐hoop (Protocol 1), adapted hula‐hoop (Protocol 2), eccentric cycling (Protocol 3), and ramp to failure (Protocol 4), applied to two NRDs, one preformed and one in situ curing.

**Methods:**

Nucleus material was removed from 40 bovine tail intervertebral disks. A NRD was inserted posteriorly into each cavity and the disks were subjected to one of four expulsion protocols.

**Results:**

NRD response was dependent on both the NRD design and the loading protocol. Protocol 1 resulted in higher migration and earlier failure rates compared to Protocol 2 in both NRDs. The preformed NRD was more likely to migrate when protocols incorporated rotation. The NRDs had equal migration (60%) and expulsion (60%) rates when using unilateral bending and ramp testing. Combining the results of multiple tests revealed complimentary information regarding the NRD response.

**Conclusions:**

Adapted hula‐hoop (Protocol 2) and ramp to failure (Protocol 4), combined with fluoroscopic analysis, revealed complimentary insights regarding migration and failure risk. Therefore, when adopting the surgical approach and animal model used in this study, it is recommended that NRD performance be assessed using both a cyclic and ramp loading protocol.

## Graphical abstract

Current nucleus replacement device (NRD) testing standards do not include a specific expulsion test as a requirement. This has resulted in multiple methods being developed inhibiting comparison between designs. This in vitro study compared four expulsion testing protocols reported in previous studies. Results demonstrated that it is beneficial to utilize a range of tests to assess NRDs at the pre‐clinical stage so that more accurate assessments of expulsion risk can be made.

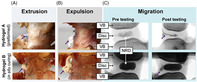

## 1 INTRODUCTION

When conservative treatments fail, discectomy or discectomy combined with fusion are the most common surgical procedures used to treat chronic low back pain.^1^, ^2^ Long‐term outcomes for both interventions are poor, with 2%–42% of fusion patients developing adjacent segment disease within 5–20 years of their original surgery,^3^, ^4^, ^5^, ^6^, ^7^ and 1%–27% of discectomy patients experiencing reherniation within 1 week to 5 years.^8^, ^9^, ^10^ Nucleus pulposus (NP) replacement, namely, the augmentation or replacement of NP material with a nucleus replacement device (NRD), has therefore been developed as an alternative treatment potentially capable of restoring disk biomechanics and alleviating pain.^11^, ^12^

NRDs can be categorized into two groups: preformed and in situ curing. Preformed NRDs enable accurate control over viscoelastic properties but typically require a large annulus (AF) incision prior to implantation.^13^, ^14^, ^15^ In situ curing NRDs can be implanted using a relatively small incision and are designed to conform to post‐surgical cavities.^11^, ^14^, ^16^ Currently, neither approach is routinely used in the clinic,^11^, ^13^, ^14^, ^15^, ^17^, ^18^, ^19^, ^20^, ^21^, ^22^, ^23^, ^24^, ^25^, ^26^, ^27^ likely due to the high risk of complications, which include device displacement and subsidence, NRD migration or expulsion through the annular implantation route, and loss of disk height.^11^, ^13^, ^28^, ^29^, ^30^, ^31^, ^32^ A wide range of pre‐clinical and in vitro mechanical tests have therefore been employed to assess and mitigate the risk of device failure via NRD expulsion; however, there is no consensus regarding the most appropriate protocol. Protocols to assess NRDs commonly include material characterization and in vitro uniaxial compression of cadaveric specimens,^33^, ^34^, ^35^, ^36^, ^37^, ^38^, ^39^, ^40^, ^41^, ^42^, ^43^, ^44^, ^45^ although more physiologically relevant complex loads, such as bending, have also been applied.^33^, ^44^, ^46^, ^47^, ^48^, ^49^, ^50^, ^51^

Notable complex loading experiments include those by Heuer et al.,^52^ who subjected bovine specimens to the hula‐hoop protocol developed by Wilke et al.^53^ The hula‐hoop setup involves continuous axial rotation of specimens (360°/min) while simultaneously applying offset cyclic loads (100–600 N). Lin et al.^36^ adapted this hula‐hoop test by rotating bovine specimens at 15° increments between +135° and −135° with respect to the annular incision and subjecting them to 1 min of cyclic loading after each rotation; a complimentary approach has also been to bend specimens by 5° and apply compression at 2 mm/min.^34^, ^36^ Meanwhile, Bao et al.^54^ and Ordway et al.^55^ assessed the risk of NRD expulsion by subjecting human cadaveric lumbar specimens to 100 000 offset loading cycles (2.5–7.5 Nm), while Christiani et al.^56^ tested porcine specimens using lateral bending, ramping an offset load to failure. The relative usefulness of one protocol over another to assess NRD expulsion risk has not been comprehensively studied, while comparisons between NRDs across studies are challenging due to the methodological differences described.

The standardized test procedures for NRD assessment outlined by ISO 18192‐2 and American Society for Testing and Materials (ASTM) 2789^57^, ^58^ provide some guidance in terms of which general methods for assessing device expulsion could be employed, but detailed parameters are not provided. This study therefore compares four expulsion testing protocols; Protocol 1: the hula‐hoop test of Heuer et al.,^52^ Protocol 2: the adapted hula‐hoop test of Lin et al.,^36^ Protocol 3: the eccentric loading used by Bao et al.^54^ and Ordway et al.,^55^ and Protocol 4: the ramp test developed by Christiani et al.,^56^ the aim being to recommend a test workflow against which future NRDs can be benchmarked. NRD migration, extrusion, and expulsion rates were assessed in response to each protocol using one preformed and one in situ NRD to provide additional context at the device design stage.

## 2 METHODS

**FIGURE 1:**
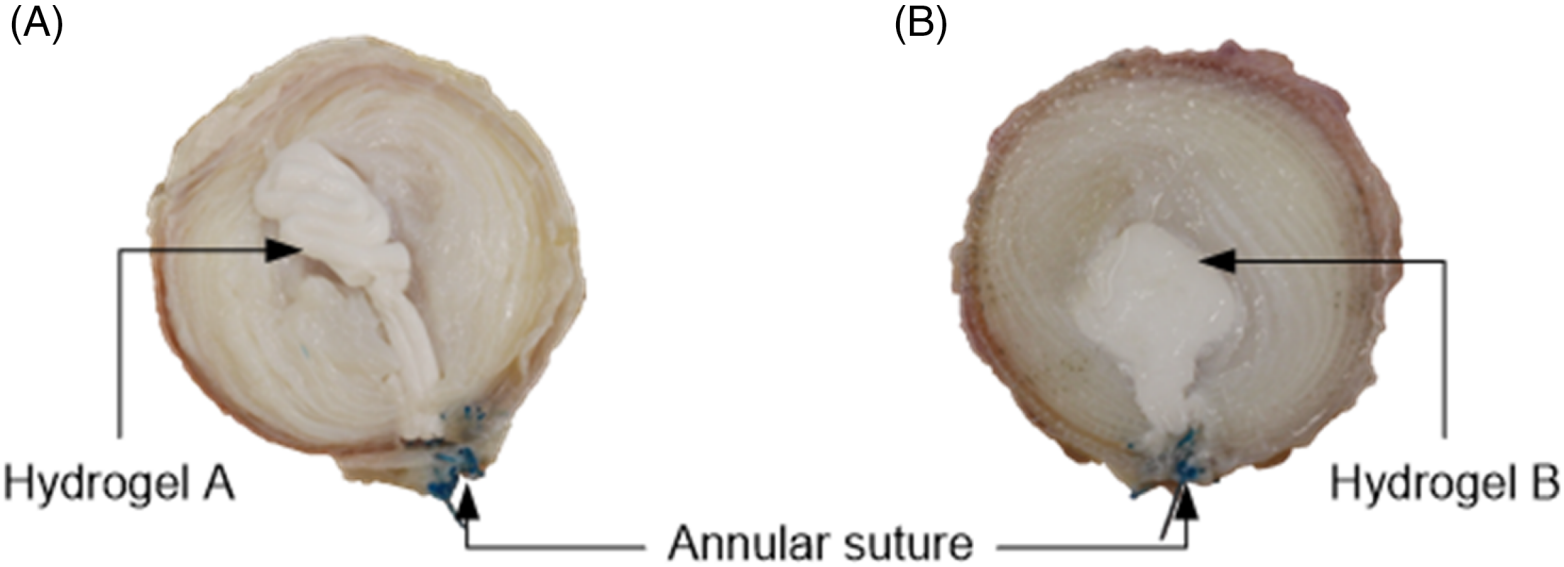
Axial view of transversely cut bovine disks following insertion of (A) Hydrogel A and (B) Hydrogel B. Hydrogel A was a thin, cylindrical (1.5 mm diameter) preformed nucleus replacement device (NRD) that bundled to fill the disk cavity. Hydrogel B was an in situ curing NRD that, once injected, conformed to fill the disk cavity prior to curing.

Two hydrogel‐based NRDs primarily consisting of polyvinyl alcohol and polyvinyl pyrrolidone were investigated. Hydrogel A was a preformed, string‐like NRD of 1.5 mm diameter, previously developed by Synthes Spine LLP (USA). It was prepared at Imperial College London according to the formulation disclosed in the granted US Patent (US 8118874 B2) under the guidance of NS. Hydrogel B was a modified, thermo‐setting form of Hydrogel A that incorporated polyethylene glycol^59^ and was provided by EB. This NRD was in situ curing and conformed to the disk cavity (Figure 1).

Different terminologies have been used to describe the failure of NRDs due to internal migration or expulsion from the disk. In this study, migration, considered a precursor to failure, was defined as either internal movement of the device or when the NRD began visibly protruding from the disk cavity (by less than 2 mm out of the site of herniation). NRD failure was classified as device extrusion (between 2 and 10 mm of NRD material seen outside the disk) or expulsion (more than 10 mm of NRD material seen outside the disk), a more severe form of failure.

### 2.1 Specimen preparation

Forty bovine segments were harvested from 15 tails obtained from a local abattoir. Three motion segments were obtained from each tail by transversally cutting the first four caudal vertebral bodies (VBs) at mid‐height. Each specimen was inspected for damage and discarded if compromised. Soft tissues were removed, segments were double bagged, and these were stored at −20°C. Cranial and caudal VBs were embedded in polymethylmethacrylate so that each intervertebral disk (IVD) was positioned centrally within a compression pot. Specimens were thawed overnight at 4°C prior to testing. Hydration was maintained through periodic spraying of 0.15 M phosphate buffered saline (PBS).

### 2.2 Nucleus replacement device material characterization

Cloyd et al.^60^ used a uniaxial compression test to characterize the unconfined Young's modulus of human NPs. This approach was replicated for both NRDs. Five samples from each hydrogel were prepared inside a 16‐well plate and cut into cylinders using an 8 mm diameter biopsy punch. Three height and diameter measurements were taken for each sample using calipers, and the averages were calculated. Using a materials testing machine (Model 5866, Instron, UK), samples were uniaxially compressed to failure at 5%/s while submerged in room‐temperature PBS. Average moduli were calculated at increments of 5% up to 25% strain. To replicate Cloyd et al.'s^60^ analysis, stress–strain curves were fit according to *σ* = *A*e^*βε*−1^ using GraphPad Prism (v. 9.4.1, GraphPad Software, LLC, USA), where *A* and *β* were average constants obtained from the model. The toe and linear region moduli were calculated at 0% and 20% strain using the tangent to the experimental curve.

### 2.3 Nucleus removal

Nuclectomy was performed using an automated shaver (Nucleotome®, Clarus Medical, USA). An ‘x’ shaped incision was made at mid‐height of the dorsolateral side of the AF using a #11 blade. A guidewire (1.5 mm in diameter) was pushed through the incision until a drop in resistance was felt, indicating that the guidewire had reached the NP. Fluoroscopic images (Fluoroscan® InSight™ FD Mini C‐Arm Imaging System, Hologic, USA) in the sagittal and coronal planes confirmed guidewire positioning. A trephine (3 mm diameter) was pushed over the guidewire to enlarge the incision so that the automated shaver's probe (3 mm diameter) could be inserted. The probe was then continuously rotated and manually moved in a fan‐like pattern to maximize NP material removal. Tissue was aspirated until no NP material passed through the tubing for 2 min, an approach used previously.^61^ Excised NP material was dehydrated for 48 h and then weighed.

### 2.4 Nucleus replacement device implantation

**FIGURE 2:**
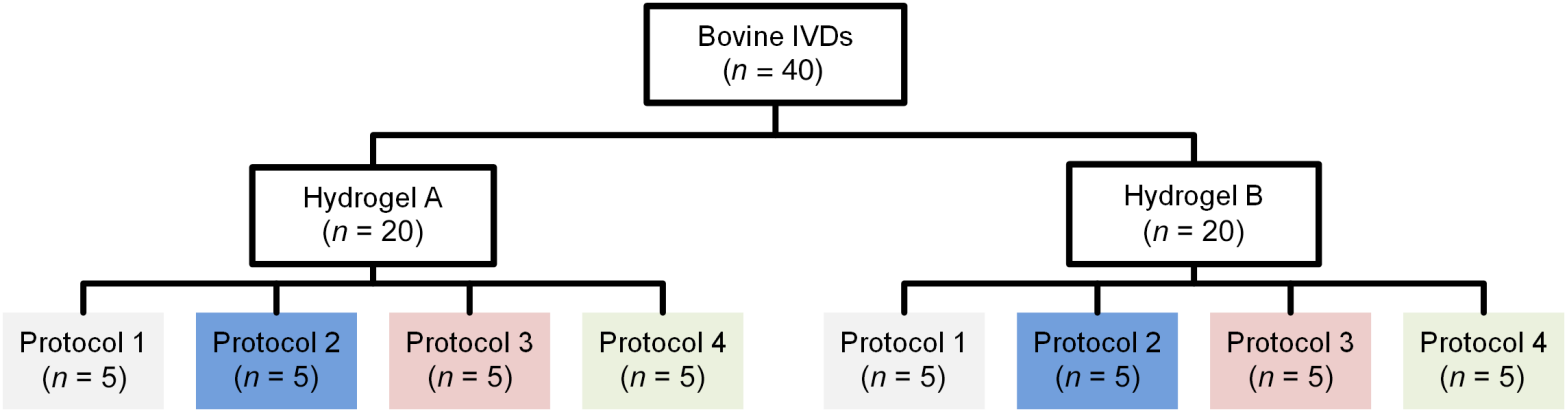
Schematic showing the different testing subgroups. Protocol 1: hula‐hoop; Protocol 2: adapted hula‐hoop; Protocol 3: unilateral cyclic loading; and Protocol 4: ramp to failure.

Following nuclectomy, specimens were assigned to Hydrogel A or Hydrogel B (*n* = 20 per group), with care being taken to assign the largest remaining disk for each tail to a different group, thereby avoiding the introduction of size‐related experimental artifacts. The average height of each disk was measured using the fluoroscopic images, while the average cross‐sectional area of each disk was calculated from two radial measurements (anterior–posterior and lateral width) taken using digital calipers with the assumption that each bovine disk was perfectly circular. Hydrogel A was cut into pre‐measured 40 mm lengths and inserted until no more could be delivered without extrusion. Any protruding material was cut and measured so that the amount of inserted material could be calculated. For Hydrogel B, a syringe was used to deliver the NRD, with the quantity of material injected determined using haptic feedback and recorded using the gage on the barrel of the syringe.^62^ This involved delivering material into the cavity until internal resistance was felt and material was visibly extruded through the annulotomy. Extruded material was again removed, and fluoroscopic images were used to confirm that no NRD material remained in the annulotomy tunnel. Previous studies have used a similar technique.^33^, ^34^, ^37^, ^41^, ^43^, ^45^, ^50^, ^63^, ^64^ All AF defects were approximately 3 mm in diameter and no noticeable increase in defect size was seen as a result of the insertion of either NRD. All AF incisions were closed using eight interrupted “U” sutures, as described in Heuer et al.^52^ Preliminary tests demonstrated that, without the AF suture, a single off‐axis compression caused almost instantaneous extrusion. Following NRD insertion, specimens were further divided into four subgroups (*n* = 5), again ordered by disk height, with each subgroup assigned a different expulsion testing protocol (Figure 2).

### 2.5 Expulsion testing

**FIGURE 3:**
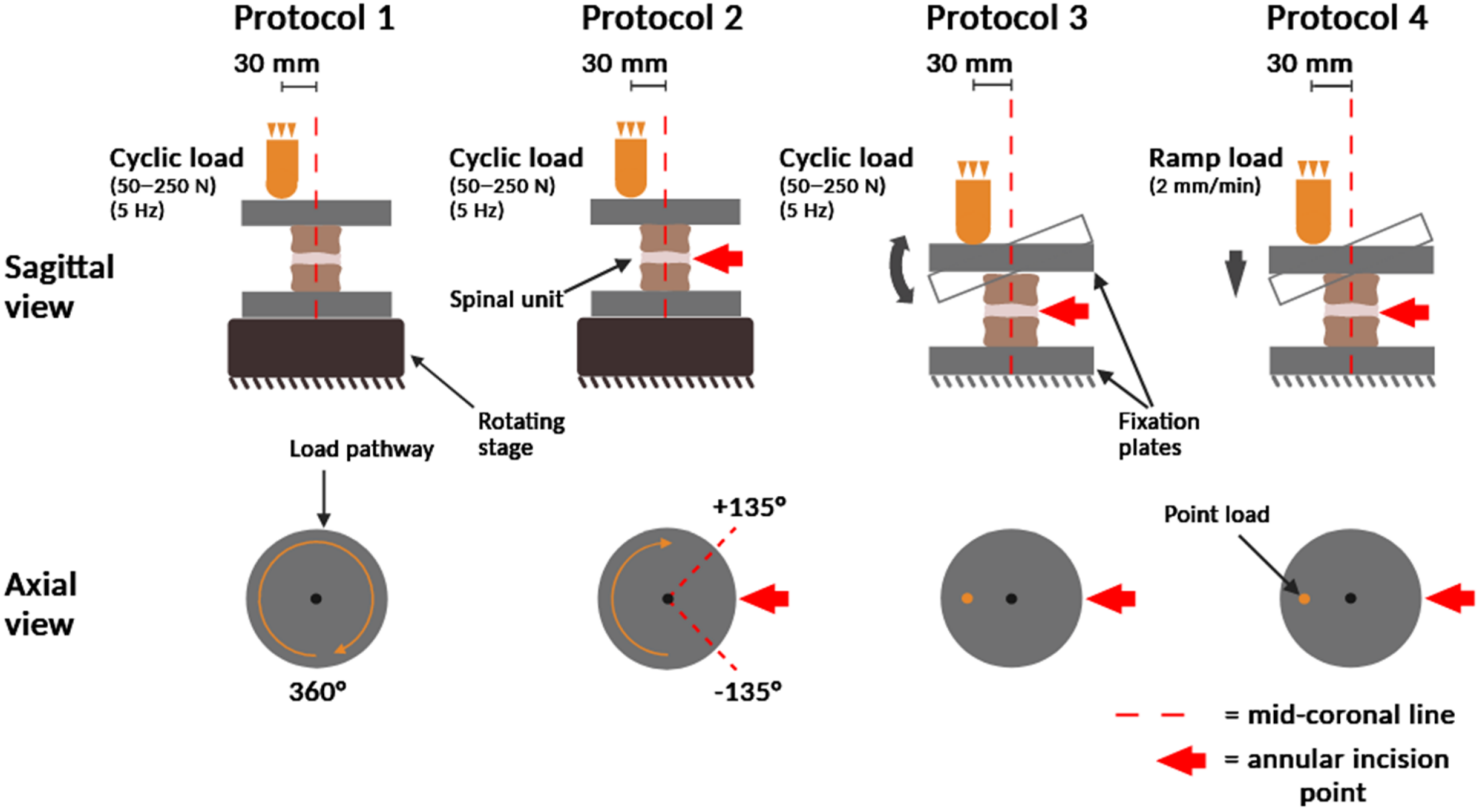
Schematic depicting the four expulsion testing protocols investigated. In all protocols, an eccentric load was applied at a moment arm of 30 mm from the coronal midline of the disk. In Protocols 1, 2, and 3, a sinusoidal load between 50 and 250 N was applied at 5 Hz until 100 000 cycles were completed or nucleus replacement device extrusion or expulsion occurred. In Protocol 4, an off‐axis ramp to failure was applied at 2 mm/min.

The four protocols that were employed to assess NRD failure risk are depicted in Figure 3.

### 2.6 Constant protocol parameters

Where possible, parameters were kept constant between protocols. All expulsion tests were carried out using a servo‐hydraulic materials testing machine (Model 8872; Instron, USA) and loads were measured using the inbuilt crosshead load cell. In all protocols, loading was applied eccentrically at an offset of 30 mm from the coronal midline of the disk, here defined as half the coronal disk width obtained using caliper measurements. A 30 mm offset has been previously used to bend human specimens to typical ranges of motions,^65^, ^66^ and preliminary work showed that 250 N applied at a 30 mm offset resulted in intact bovine specimens exhibiting rotations similar to the extension values reported for humans^65^, ^67^, ^68^ (16.9 ± 0.4° vs. 16.6 ± 2.3°). These were small enough that specimens could be subjected to multiple loading cycles before failure occurred, and this offset was therefore applied in all protocols.

For the cyclic loading tests (Protocols 1, 2, and 3), a sinusoidal load ranging between 50 and 250 N was applied at 5 Hz. The tests were terminated once 100 000 cycles were completed or if failure occurred.^36^ Physiologically, 100 000 cycles at 5 Hz approximates to 3–10 months of loading in the human spine,^69^ and the maximum number of cycles was set so that tests were complete within 12 h, limiting the impact of specimen deterioration.^70^ The outcome measure of the tests described in Protocols 1, 2, and 3 was cycles‐to‐failure, while for Protocol 4 it was bending angle at failure.

### 2.7 Protocol 1: hula‐hoop test

A custom‐built rig consisting of a rotating stage (MM‐Engineering GmbH, Germany) and fixation plates were used to continuously rotate specimens at 360°/min while eccentrically and cyclically compressing them.

### 2.8 Protocol 2: adapted hula‐hoop test

The same setup described in Protocol 1 was used, except specimens were rotated in increments of 15° between +135° and −135° from the axis opposite the annular incision. At each increment, 1 min of cyclic loading was applied.

### 2.9 Protocol 3: cyclic bending test

Specimens were positioned such that eccentric loads were cyclically applied antipodal to the insertion site.

### 2.10 Protocol 4: ramp test

An eccentric load was applied opposite the insertion site at 2 mm/min.^34^, ^36^, ^71^ The test continued until extrusion occurred or a geometric constraint was met. A digital camera (EOS 750D, Canon, Japan) captured one image per second. An open‐source Digital Image Correlation tool (DICe, Sandia Corporation, USA) was used to calculate rotation angles.

### 2.11 Disk height measurements

Sagittal and coronal plane fluoroscopic images of specimens were captured at each stage; intact, post‐nuclectomy, post‐treatment with NRD, and post‐test. A calibration stick was used so that mid‐disk heights could be quantified in ImageJ (v. 1.53t, National Institutes of Health, USA). Fluoroscopic images were also used to identify device migration.

### 2.12 Statistical analysis

All statistical tests were conducted using GraphPad Prism with a significance level of *p* < 0.05. A two‐way analysis of variance was used to compare NRD Young's modulus to the native NP values reported by Cloyd et al.^60^ and to compare the cycles to failure data between protocols. A Shapiro–Wilk test was performed to confirm the normality of the disk dimensions and cycles to failure data. Paired *t*‐tests were used to assess disk height changes relative to their intact state, and to investigate the relationship between the volume of NP removed and NRD inserted. Correlations were established using Pearson's *r* correlation analysis.

## 3 RESULTS

### 3.1 Material properties of nucleus replacement devices

**FIGURE 4:**
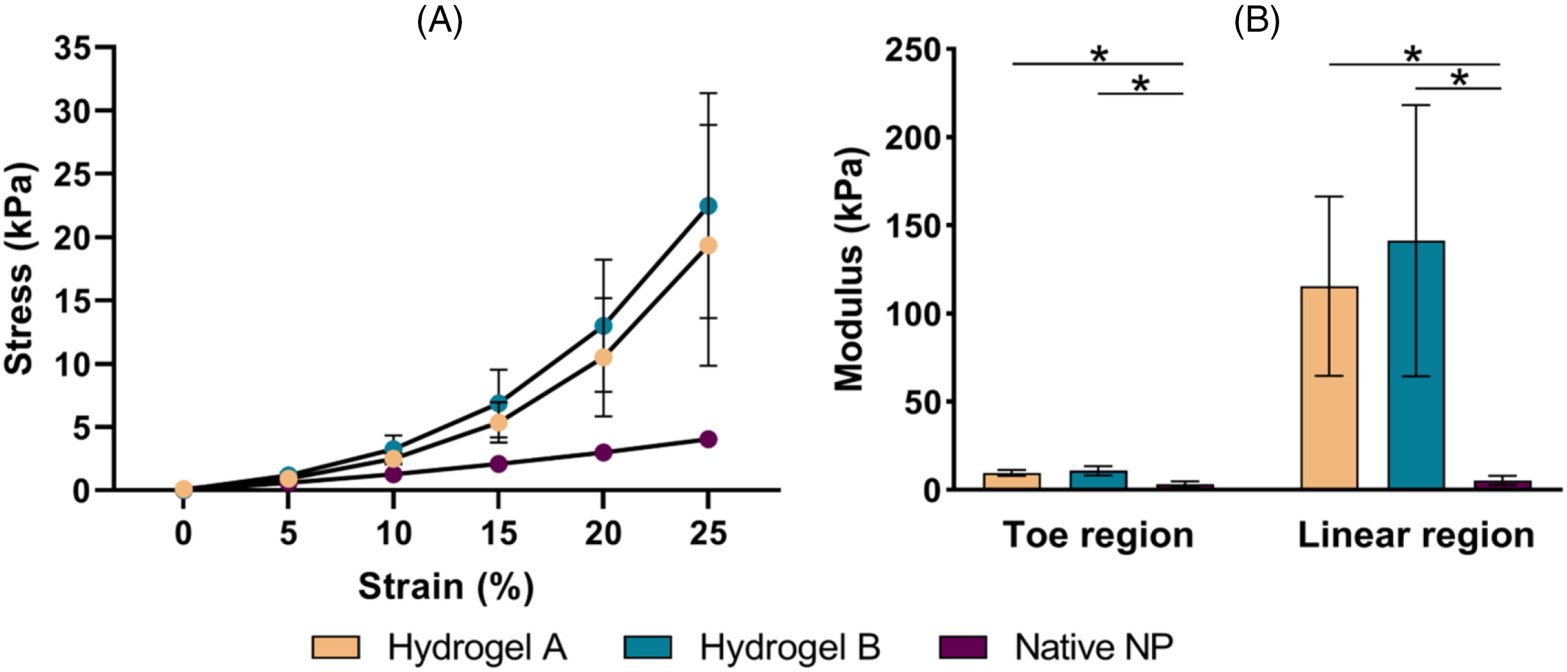
Unconfined compression results from Hydrogel A (preformed) and Hydrogel B (in situ curing) compared to the human cadaveric NP values reported by Cloyd et al.^60^ (A) Average stress–strain curves 20 obtained using mean hydrogel values fit according to *σ* = *A*(e^*βε*−1^). (B) Average toe and linear region Young's moduli. Error bars represent one standard deviation. Significant differences are denoted by an asterisk (*p* < 0.05).

Average stress–strain responses for Hydrogel A and Hydrogel B were modeled by *σ*_A_ = 2.53e^12.82*ε*−1^ and *σ*_B_ = 2.18e^12.04*ε*−1^ (Figure 4A, Pearson's *r* ^2^ > 0.992), respectively. There were no significant differences between the toe and linear region moduli of the NRDs (*p* ≥ 0.417). However, both devices were significantly stiffer in both regions compared to native NP^60^ (Figure 4B, *p* ≤ 0.004).

### 3.2 Mass of material removed from the disk and volume of nucleus replacement device inserted

On average, 0.104 ± 0.043 g of dry NP material was removed during nuclectomy. On average, 52.3 ± 22.6 mm of Hydrogel A was inserted into the disks, corresponding to a volume of 0.092 ± 0.040 cm^3^. This was approximately a factor of 10 below the 0.958 ± 0.538 cm^3^ of Hydrogel B that was delivered. No correlation was observed between NP mass removed and NRD volume inserted, regardless of whether the NRD was preformed (Pearson's *r* = 0.025, *p* = 0.932) or in situ curing (Pearson's *r* = 0.403, *p* = 0.086).

### 3.3 Nucleus replacement device failure

**FIGURE 5:**
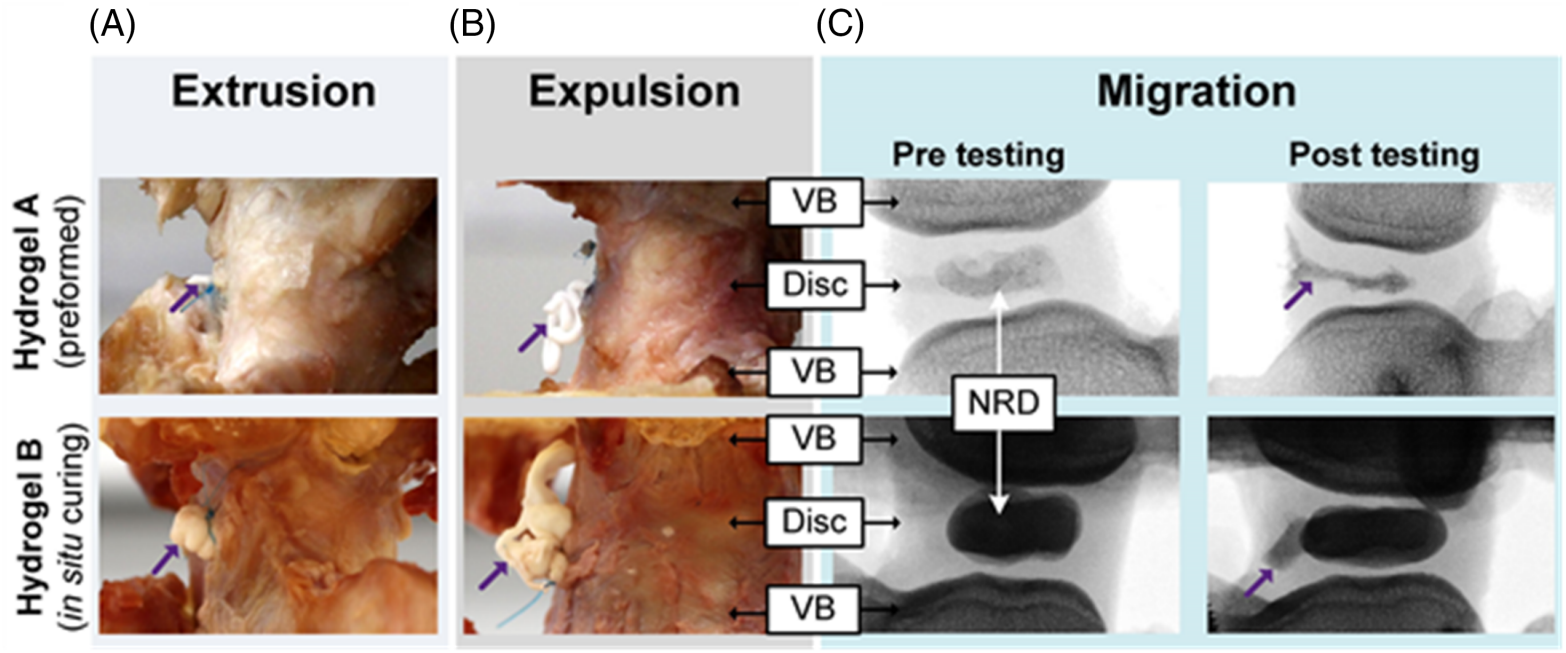
Images of bovine specimens following (A) extrusion, (B) expulsion, and (C) migration. The purple arrows point to nucleus replacement devices (NRDs) following failure. NRD migration was observed using fluoroscopic images (C). Extrusion was defined as NRD protrusion exceeding 2 mm through the annular defect, while expulsion was a protrusion of more than 10 mm. Migration was defined as an observable geometric change or internal displacement of the NRD, plus minor protrusions (less than 2 mm) not meeting the criterion for failure via extrusion or expulsion. VB, vertebral bodies.

Figure 5 shows photographs and fluoroscopic images of extrusion, expulsion, and migration.

**FIGURE 6:**
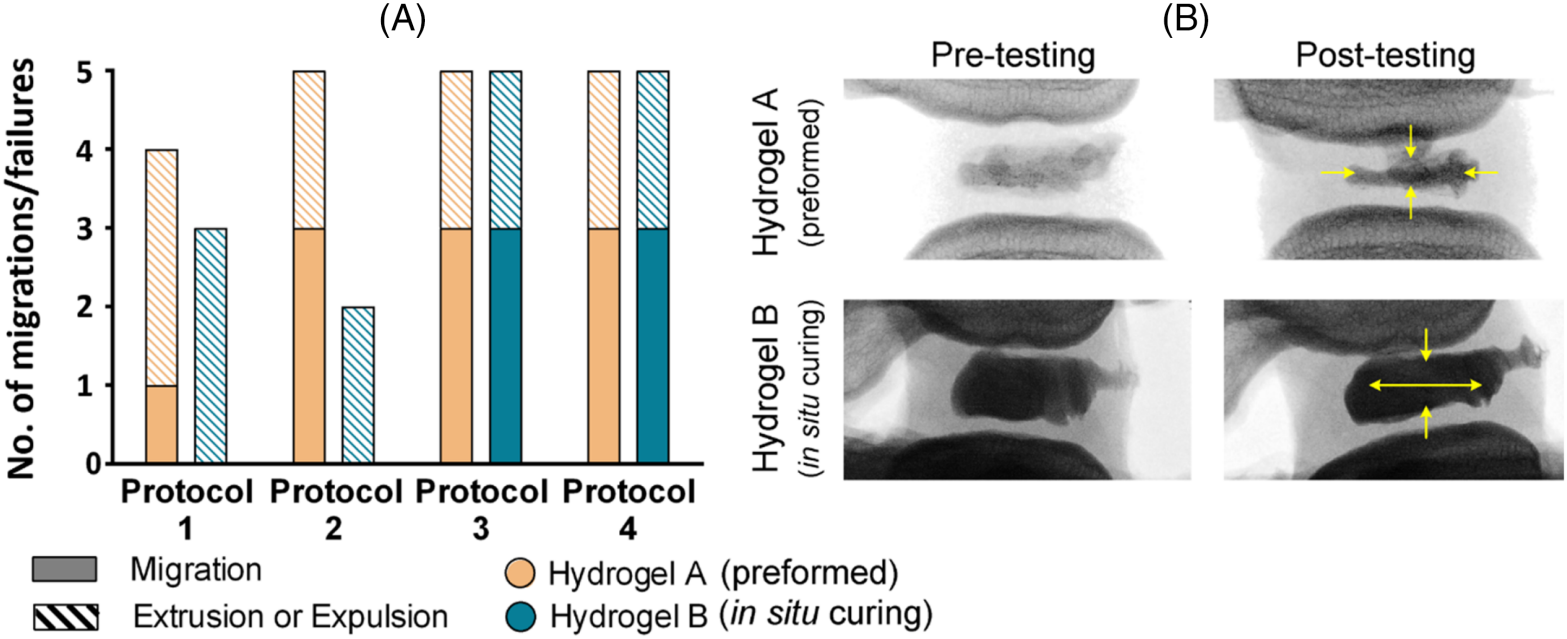
(A) Bar graph summarizing the effects of using different loading protocols on the two nucleus replacement devices (NRDs). (B) Lateral view fluoroscopic images of two typical instances of NRD geometry change. The yellow arrows indicate the direction in which the NRD shape has changed. *n* = 5 of each hydrogel was tested for each protocol.

Figure 6A shows the failure rates and failure modes for all specimens. Hydrogel A appeared to be better able to resist migration under Protocol 1 compared to other protocols. Hydrogel B was more resistant to migration in both Protocols 1 and 2 compared to Protocols 3 and 4. Combining results from all protocols shows that the in situ curing Hydrogel B was 20% less likely to migrate compared to the preformed Hydrogel A (*p* = 0.356). Rates of NRD failure were identical using Protocols 3 and 4, with differences in NRD response highlighted using a combination of cyclic and ramp tests. Fluoroscopic images revealed internal geometric NRD changes, considered a precursor to failure (Figure 6B).

### 3.4 Cycles to failure (Protocols 1, 2, and 3)

**FIGURE 7:**
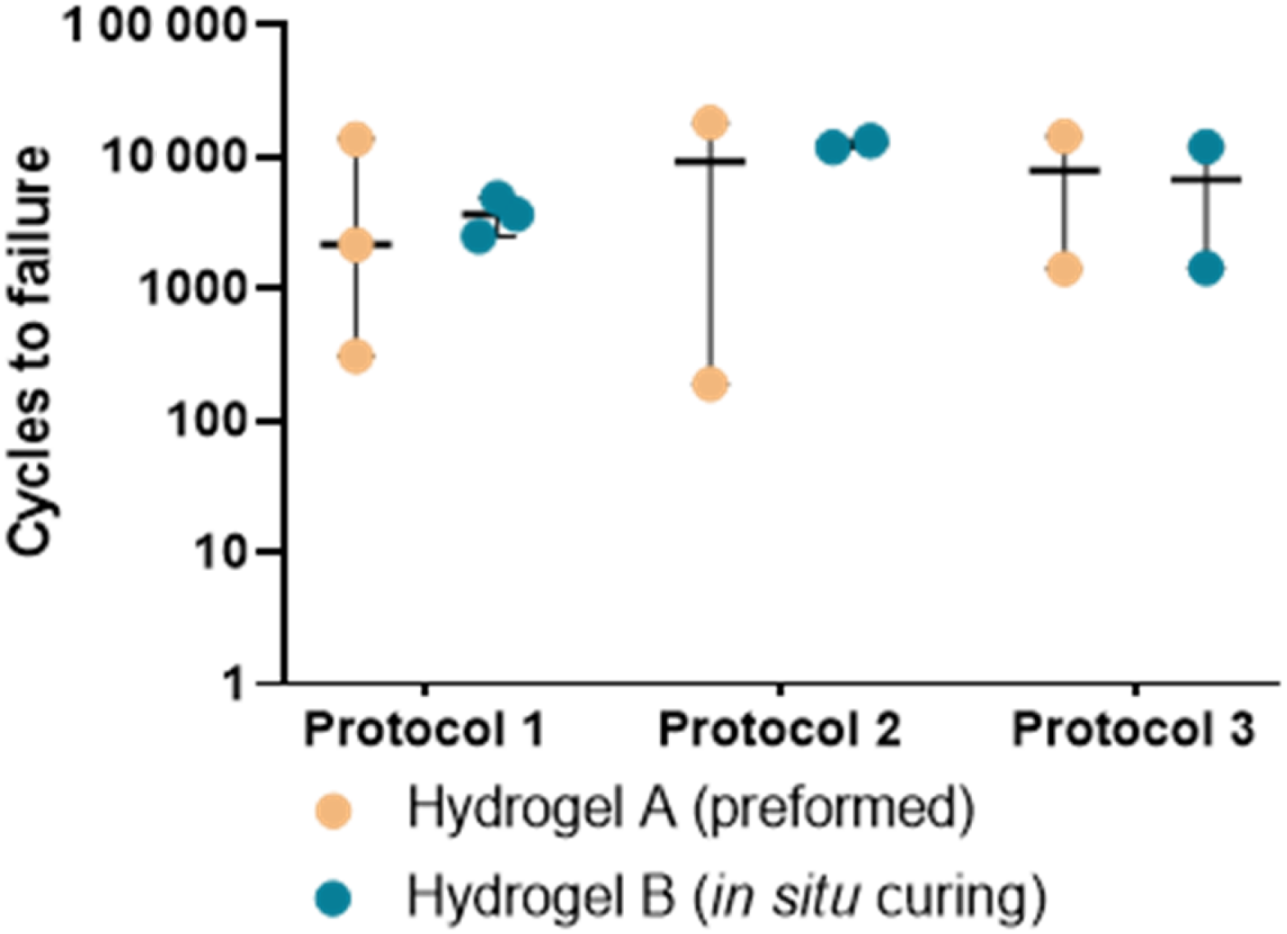
Logarithmic dot‐plot of the cycles to nucleus replacement devices extrusion or expulsion (note data from tests where migration was seen are not included here) when using Protocols 1, 2, or 3. Median values are reported with bars spanning the range.

Failure due to extrusion or expulsion occurred within 19 000 cycles (range 190–18 249) across all protocols (Figure 7). Protocol 1 resulted in an earlier failure rate for both NRDs compared to Protocols 2 and 3. Grouping data from all protocols, Hydrogel A failed in 7294 ± 7079 cycles. This was similar to Hydrogel B, which failed in 7085 ± 4690 cycles (*p* = 0.976).

### 3.5 Ramp to failure: peak load and angle (Protocol 4)

Three specimens from each hydrogel met their geometrical constraints, leaving only two samples that could ramp to failure. This highlights the importance of pot design when ramping to ranges of motion past physiological limits, particularly when using bovine disks. Of the failed samples, mean axial failure angle for the preformed NRD was 21.2°, while for the in situ curing NRD it was 20.4°. The geometrical constraints were met on average at 25.7° and 21.9°, respectively.

### 3.6 Intact cross‐sectional areas and disk height changes

**FIGURE 8:**
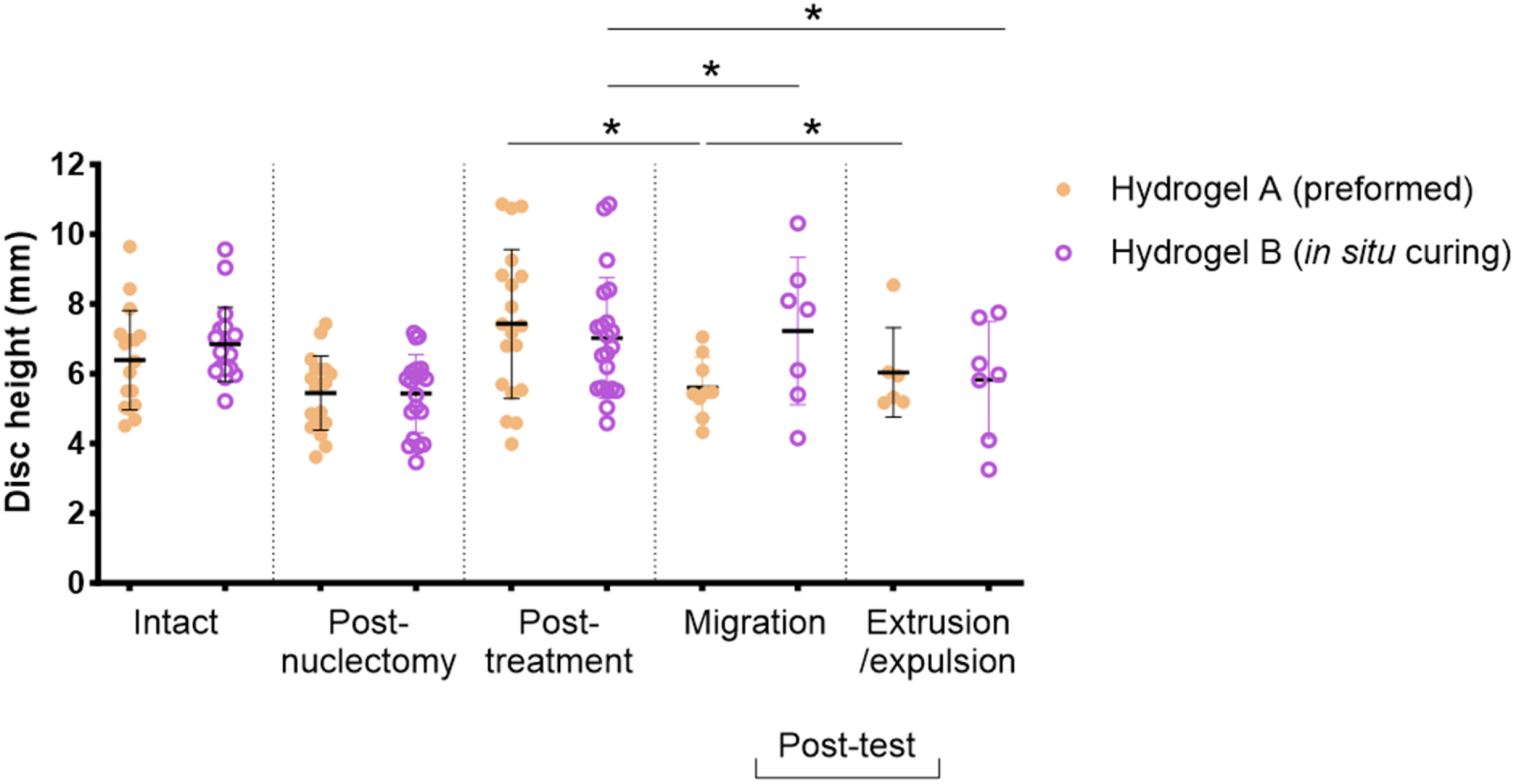
Dot‐plot graph of intervertebral disk heights at the various stages of testing. Significant differences are denoted by asterisks (*p* < 0.05).

Average cross‐sectional area of all intact disks was 467.6 ± 80.3 mm^3^. Average intact disk height was 6.6 ± 1.2 mm. Dimensional data for all intact groups and subgroups was normally distributed with no outliers. There was no significant difference between the intact disk heights used for tests involving either Hydrogel A or Hydrogel B, nor across subgroups. Disk height was significantly reduced following nuclectomy (19.2%, *p* < 0.001) compared to intact; however, there was no significant difference between disk heights post‐nuclectomy (Figure 8). There was no statistical difference between the height disks were restored to by either Hydrogel A or Hydrogel B (*p* = 0.089), nor were these disk heights significantly different from the intact disks in either category. Analysis post‐test indicated that when migration specifically occurred, Hydrogel A disk height fell significantly 24.5% below post‐treatment height (*p* = 0.0196) while for Hydrogel B disk height was increased by 2.8%; this change was not significant (*p* = 0.9580). When failure occurred, height loss for Hydrogel A fell by 18.7% although not significantly (*p* = 0.1196), while for Hydrogel B, it fell significantly, by 17.1% (*p* = 0.0488).

## 4 DISCUSSION

Collectively, the data indicates that combining multiple protocols during the pre‐clinical NRD development stage is likely to reveal a more complete picture of NRD expulsion risk than when tests are conducted in isolation. A combination of cyclic (Protocols 1, 2, and 3) and non‐cyclic (Protocol 4) tests also revealed that, while the response of the preformed and the in situ NRD were broadly similar, there were differences in their behavior that were best brought out when the results of different tests were combined and novel fluoroscopic image analysis was used to assess migration.

When deployed in isolation, each protocol has undoubted merits. For example, complex loading protocols such as Protocols 1 and 2 enable NRD expulsion to be investigated without forcing device migration in any one direction. This might allow better prediction of in vivo performance than similar cyclic protocols employing unilateral loading, such as Protocol 3, which provides direct guidance on the number of unilateral cycles to failure but at the cost of potentially forcing migration toward the annulotomy. Protocol 4, in contrast, while also unilateral in nature, provides other specific information regarding expulsion risk; if peak loads extend beyond a physiological range, it is possible to infer that the NRD has the potential to resist extrusion in vivo.

In this study, the high migration and failure rates and the lower cycles to failure in Protocol 1 compared to Protocol 2 (4533 ± 4314 and 10 893 ± 6620 cycles (Figure 7), respectively) (*p* = 0.406) suggest that Protocol 1 may have resulted in more extreme loading. This protocol applied a continuous cyclic load around the entire IVD, which is not physiologically relevant to humans. Human disks are not circular and their rotational stiffnesses differ substantially in flexion and extension. Specimen hyperflexion is a plausible explanation for the lower number of cycles to failure observed using Protocol 1, even in bovine tails, and, hence, it can be tentatively suggested that Protocol 1 is both more invasive and less physiologically relevant than Protocol 2. As a result, Protocol 2 provides a better indication of likely in vivo performance, since it additionally allows the targeting of precise areas of human geometry (e.g., posterolateral corners), even though aspects of Protocol 2 are also not physiological.

In contrast, three out of five specimens migrated when using either Protocols 3 or 4, irrespective of NRD type (Figure 6A). Considering the difference in NRD volume between Hydrogel A and Hydrogel B, this was unexpected. String‐like, preformed NRDs can be advantageous since they reduce the likelihood of the NP cavity being overfilled; however, this comes at the cost of introducing gaps within the bundled material (Figure 1) and limiting the relative volume of material that can be inserted in comparison to in situ alternatives; such gaps can increase the risk of subsidence and expulsion.^13^, ^17^, ^31^ Voids within the string‐like preformed NRDs may also lead to uneven load distributions, adversely affecting IVD biomechanics and treatment efficacy, whereas in situ curing NRDs have the potential to completely fill the NP cavity (Figure 1), avoiding this limitation. Fully filled, but not overfilled, cavities are likely to result in a more uniform distribution of loading, assuming the geometry of the cavity is approximately symmetrical and homogeneous NP removal has been achieved, for example, using an automated shaver.^61^ It was therefore notable that Hydrogel A demonstrated higher rates of migration than Hydrogel B when using Protocols 1 and 2 (20% and 60% higher, respectively); the implication is that the continuous rotation of the base plate meant that the fully confined in situ curing NRD was not forced in any one direction by Protocols 1 and 2, while the loosely constrained preformed NRD was able to move (Figure 6A). In contrast, migration rate appeared to be independent of NRD type or inserted volume when using Protocols 3 and 4, most likely because of the unilateral application of force. Therefore, despite similarities between Protocols 1, 2, and 3, and between Protocols 3 and 4, different information can be gleaned from each.

Care was taken to replicate the published methodologies while allowing comparability between NRD responses in the current study. As a result, however, the data generated were not always comparable with those from previous studies. For instance, Protocol 1 had a maximum cycle number of 13 656, almost double the 7996 cycles reported by Heuer et al.^52^ This was possibly due to more extreme loading (24 vs. 7.5 Nm) or because different NRDs were investigated (collagen matrix‐based device of unreported stiffness). Protocol 2's maximum number of cycles was also higher than Lin et al.'s^36^ (18 249 vs. 13 000), even though their study used a smaller moment (6 Nm). This could be due to their lack of an AF closure system, or because the NRD investigated was approximately four times less stiff than the one used here (37.8 ± 4.6 vs. 141.4 ± 76.9 kPa). In contrast, specimens from Protocol 3 failed within 14 384 cycles while all specimens tested by Bao et al.^54^ and Ordway et al.^55^ completed 100 000 cycles without signs of migration. Bao et al.^54^ and Ordway et al.^55^ tested at a slower frequency (2 vs. 5 Hz), a smaller offset (10 vs. 30 mm), used human rather than bovine specimens, and evaluated a different NRD (Nubac™, Pioneer Surgical Technology, USA); Nubac™ is an articulating intradiscal NRD similar to a total disk replacement in that it fixes to vertebrae. This possibly explains Nubac's superior ability to resist expulsion and migration, although it is also possible that bovine tails are less able to withstand such an invasive protocol than human disks. Lastly, when using Protocol 4, 40% of specimens failed due to expulsion (Figure 6A), while Christiani et al.^56^ did not observe any expulsions despite using a similar ramp rate (estimated from an angle/time to failure calculation), 0.10°/s vs. 0.14°/s, respectively. This discrepancy is likely due to their smaller offset (25.4 mm), use of porcine rather than bovine specimens, a smaller annular incision (1.02 mm diameter puncture vs. 3 mm), and an NRD with different stiffness. As multiple factors seem to affect expulsion rate, it is therefore recommended that NRD testing closely recreates the clinical scenario by using the same AF incision size, AF closure system (if any), and NRD insertion approach as those deployed at the point of use.

In all cases of extrusion or expulsion, the NRD exited the disk between the sutures (i.e., path of least resistance, Figure 5), an observation also reported by Heuer et al.,^52^ suggesting that the formation of an inner mechanical barrier (in situ cured NRD larger than the annulotomy) did not provide a higher resistance to expulsion due to internal pressures. Pooling results from Protocols 1, 2, and 3, showed that extrusion or expulsion occurred within 19 000 cycles, which is significantly lower than the target 100 000. Interestingly, ASTM and ISO standards recommend testing to 10 million cycles^57^, ^58^; however, this is challenging when using cadaveric IVDs due to tissue degradation^59^ and previous studies have demonstrated that biological models can successfully complete 100 000 cycles.^24^, ^52^, ^53^, ^66^, ^72^, ^73^, ^74^ As it was possible to identify differences in (i) migration using fluoroscopic images, (ii) extrusion and expulsion rates, and (iii) loss of disk height, at a substantially lower number of cycles than recommended using Protocols 1, 2, and 3, it can be concluded that both NRD designs tested here require further development before they can reliably resist expulsion.

It was difficult to reach conclusions on the suitability of each NRD from the data generated using Protocol 4, as 6 of the 10 samples survived ramp testing to the geometric constraints, limiting statistical comparisons. There are therefore few insights that can be drawn from data specifically regarding failure loads, angles, and moments; however, as Figure 6 demonstrates, Protocol 4 has the potential to provide alternative information regarding NRD behavior that may complement data generated using cyclic forms of loading. This study revealed that NRD migration and failure rates were identical in both Protocols 3 and 4, indicating that offset cyclic loading and offset ramp loading resulted in similar NRD behavior. In future, with the aid of a larger sample size, Protocol 4 could be increased in sophistication by incorporating more physiological loading, for example, applying pure moments with axial preloads, and through investigations seeking to optimize ramp to failure as a form of NRD assessment, including understanding the relative effect of using load control, angle control, and different offsets.

In terms of disk height, this was reduced by nuclectomy and adequately restored by the insertion of either of the two NRDs (Figure 8). NRD failure type and rate did not correlate with disk dimensions, mass of NP removed, or volume of NRD inserted (Pearson's *r* = 0.0031–0.403, *p* > 0.05), suggesting that predicting failure based on these quantities is not possible. Interestingly, disk height for Hydrogel A was reduced significantly only when migration occurred, whereas significant disk height reductions were only observed for Hydrogel B when failure occurred. When these results are combined with the data presented in Figures 6 and 7, it becomes possible to envisage how a suite of tests could indicate which NRDs are at an increased risk of migration under certain loading scenarios, how different types of loading might result in different rates of extrusion or expulsion, and how particular tests might be especially well suited to assessing the failure risk of preformed or in situ curing devices. It is therefore recommended that a suite of tests be used to assess NRD loading responses, to more accurately assess and mitigate the likelihood of poor clinical outcomes in a physiological setting. Based on the results of this study, a combination of Protocols 2 and 4 would be most effective when assessing whether a device is more at risk of migration or failure. However, further work is required to develop these conclusions using an expanded range of NRDs.

Besides the sample size, this study is not without limitations. Firstly, it lacks a control group. Intact disks were used in preliminary studies to identify the appropriate loading parameters used in testing protocols; however, experiments using intact disks and disks following nuclectomy but prior to NRD insertion would have provided additional insight into the relative performance of the NRDs under different protocols. In a related limitation, the study deploys parameters designed to replicate loads and moments experienced within the human disks but applies these to bovine tail disks. Bovine specimens were used because of their similar biomechanical properties, swelling pressure, and heights compared to healthy human disks^75^, ^76^, ^77^, ^78^; this choice also allowed confounding variables such as degeneration, age, sex, and level to be avoided. However, bovine specimens are almost circular in nature and are physiologically adapted to a higher range of motion than human disks. Additionally, subjecting bovine specimens to a relatively high 7.5 Nm of bending to achieve human‐like loading scenarios may have exacerbated failure rates, although this level of bending is similar in magnitude to those used by other bovine lumbar disk studies that have used various loading protocols.^24^, ^52^, ^79^ The rationale for using this relatively high moment was to enable direct comparisons to be made across existing studies, and to model extreme but physiologically relevant scenarios in the knowledge that the NRDs would be unlikely to be exposed to these scenarios in humans. It is partially because of these experimental choices that Protocol 2 can be favored over Protocol 1, alongside the fact that Protocol 2 allows physiological loads to be better targeted at regions where NRD failure is most likely to occur in humans.

Future studies should utilize an expanded range of NRDs to confirm whether certain tests are better at identifying migration or failure risk for preformed or in situ NRDs. To better understand the impact of NRD volume, intrinsically linked to NRD type, it would be valuable to compare NRDs that allow an equivalent volume of material to be inserted into the disk space; this would lead to greater insight regarding the impact of NRD volume on test outcomes in the context of other factors such as device stiffness and whether the NRDs were preformed or in situ. Other recommended extensions to the study include assessing NRDs with significantly different stiffnesses or chemical compositions (e.g., elastomeric devices similar to PDN‐Hydraflex,^31^ Aquarelle,^11^ NeuDisc,^80^ DASCOR,^13^ or NuCore^21^) as these factors were similar here; in the longer term, related studies investigating the response of ex vivo cultured specimens containing cellular components could be of additional value.^81^, ^82^ As AF closure sutures were implemented, deviating from clinical practice, investigating different closure techniques would also be worthwhile. This step was needed to prevent expulsion following a single off‐axis compression; however, it is not known whether this step affected expulsion risk in either the preformed or in situ case disproportionately.

Lastly, it is advised that future studies investigate the effect of different testing parameters, such as changes to loading offset distances and magnitudes, since results were adapted from previous studies. For greater clinical relevance, human specimens could be employed, shedding greater light on the applicability of this current study and the conclusions drawn, as bovine disks are physiologically adapted to a higher range of motions than human tissues. Human specimens are therefore unlikely to be exposed to some of the more extreme conditions presented in some publications. If possible, this advanced human study would also benefit from incorporating a six‐degree of freedom load cell directly beneath the specimen, allowing both bending moments and axial loads to be measured in close proximity to the disks. These tests would need to closely follow Protocol 4, particularly in terms of the angle of bending, which should ensure rotation away from the annulotomy, while the area targeted by Protocol 2 would also need careful consideration. Additionally, these human tests would provide more information regarding the clinical translatability of data obtained from bovine specimen experiments, and whether loading protocols should be scaled or matched with the physiology of the animal model being used to better replicate the performance of NRDs in vivo.

## 5 CONCLUSIONS

NRD response was dependent on both NRD design and the loading protocol used. Multiple tests combined with fluoroscopic imaging could be deployed in tandem to better assess the risk of device failure and differences in internal migration, a precursor to disk failure. Therefore, based on these bovine experiments where NRDs were inserted posteriorly, it is recommended that implanted NRDs should not show signs of migration, extrusion, or expulsion within 100 000 cycles using Protocol 2 (adapted hula‐hoop), and that failure loads should be beyond the physiological range when assessed using Protocol 4 (ramp test). If other animal models are used, particularly those with a reduced physiological range of motion, other combinations of protocols may be optimal, but the study demonstrates that combinations of mechanical tests can contribute to improving pre‐clinical development of NRDs in the future, which will result in better outcomes during clinical trials.

## AUTHOR CONTRIBUTIONS

TR and NN designed the study. TPS lent the automated shaver. NS and EB provided the raw material for the synthesis of Hydrogel A and Hydrogel B, respectively. TR and LH analyzed the data and drafted the manuscript, which was edited by NN, MK, NS, EB, and TPS. The manuscript has been approved by all authors before submission.

## FUNDING INFORMATION

Part of this work was funded by an Imperial College Research Fellowship for Nicolas Newell and an EPSRC DTP CASE Conversion Studentship for Tamanna Rahman (EP/R513052/1) partially funded by Watson Medical Ltd. Innovate UK funded Matthew J. Kibble through a Biomedical Catalyst grant (10051703). Equipment from the Engineering and Physical Sciences Research Council (EPSRC ‐ EP/V029452/1) funded Injury and Reconstruction Biomechanics Test Suite was used as part of this study.

## CONFLICT OF INTEREST STATEMENT

Thomas P. Schaer was involved in research support as Principal Investigator and the patenting of the hydrogel‐based technologies for ReGelTec, Inc. and Johnson and Johnson DePuy‐Synthes. Additionally, Thomas P. Schaer is a paid consultant and stock supplier for ReGelTec, Inc. Nigel Smith was involved in the development and patenting of hydrogel‐based technologies for Synthes Spine as Director of Non‐Fusion Technologies. Nigel Smith and Erik Brewer were involved in the development and patenting with ReGelTec Inc. of the Hydrafil™ Injectable hydrogel for minimally invasive treatment for treating degenerative disk disease. Nigel Smith and Erik Brewer are current shareholders of ReGelTec Inc. (https://regeltec.com/) that is active in the clinical evaluation of Hydrafil™. Additionally, Erik Brewer receives research funding from ReGelTec, Inc.
